# Author Correction: Multi-institutional atlas of brain metastases informs spatial modeling for precision imaging and personalized therapy

**DOI:** 10.1038/s41467-025-60522-w

**Published:** 2025-06-02

**Authors:** Jorge Barrios, Evan Porter, Dante P. I. Capaldi, Taman Upadhaya, William C. Chen, Julian R. Perks, Aditya Apte, Michalis Aristophanous, Eve LoCastro, Dylan Hsu, Payton H. Stone, Javier E. Villanueva-Meyer, Gilmer Valdes, Fei Jiang, Michael Maddalena, Ase Ballangrud, Kayla Prezelski, Hui Lin, Jinger Y. Sun, Muhtada A. K. Aldin, Oi Wai Chau, Benjamin Ziemer, Maasa Seaberg, Penny K. Sneed, Jean L. Nakamura, Lauren C. Boreta, Shannon E. Fogh, David R. Raleigh, Jessica Chew, Harish Vasudevan, Soonmee Cha, Christopher Hess, Ruben Fragoso, David B. Shultz, Luke Pike, Shawn L. Hervey-Jumper, Derek S. Tsang, Philip Theodosopoulos, Daniel Cooke, Stanley H. Benedict, Ke Sheng, Jan Seuntjens, Catherine Coolens, Joseph O. Deasy, Steve Braunstein, Olivier Morin

**Affiliations:** 1 Department of Radiation Oncology, University of California San Francisco, San Francisco, CA USA; 2 Department of Radiation Oncology, University of California Davis Health, Sacramento, CA USA; 3 Department of Radiation Oncology, Memorial Sloan Kettering, New York, NY USA; 4 Department of Medical Physics, Memorial Sloan Kettering, New York, NY USA; 5 Department of Radiology and Biomedical Imaging, University of California San Francisco, San Francisco, CA USA; 6 Department of Neurological Surgery, University of California San Francisco, San Francisco, CA USA; 7 Department of Epidemiology and Biostatistics, University of California San Francisco, San Francisco, CA USA; 8 Moffit Cancer Center, Tampa, Florida USA; 9 Radiation Medicine Program, Princess Margaret Cancer Centre, University Health Network, Toronto, ON Canada; 10 Department of Medical Biophysics, University of Toronto, Toronto, ON Canada; 11 UCSF/UC Berkeley Graduate Program in Bioengineering, San Francisco, CA USA; 12 Department of Pathology, University of California San Francisco, San Francisco, CA USA

**Keywords:** CNS cancer, Cancer models, Metastasis

Correction to: *Nature Communications* 10.1038/s41467-025-59584-7, published online 15 May 2025

In this article the author’s name Joseph O. Deasy was incorrectly written as Joe Deasy. Second, Joseph O. Deasy was incorrectly linked to the affiliation 3 and 4, instead of just affiliation 4. The original article has been corrected.

