# Supplementary material for: Multi-institutional atlas of brain metastases informs spatial modeling for precision imaging and personalized therapy
Source: Nat Commun. 2025 May 15;16:4536. doi: 10.1038/s41467-025-59584-7 (PMC12081687; doi:10.1038/s41467-025-59584-7)
Supplement: Supplementary file 1 — Supplementary Information [file 41467_2025_59584_MOESM1_ESM.pdf]

Supplementary Information

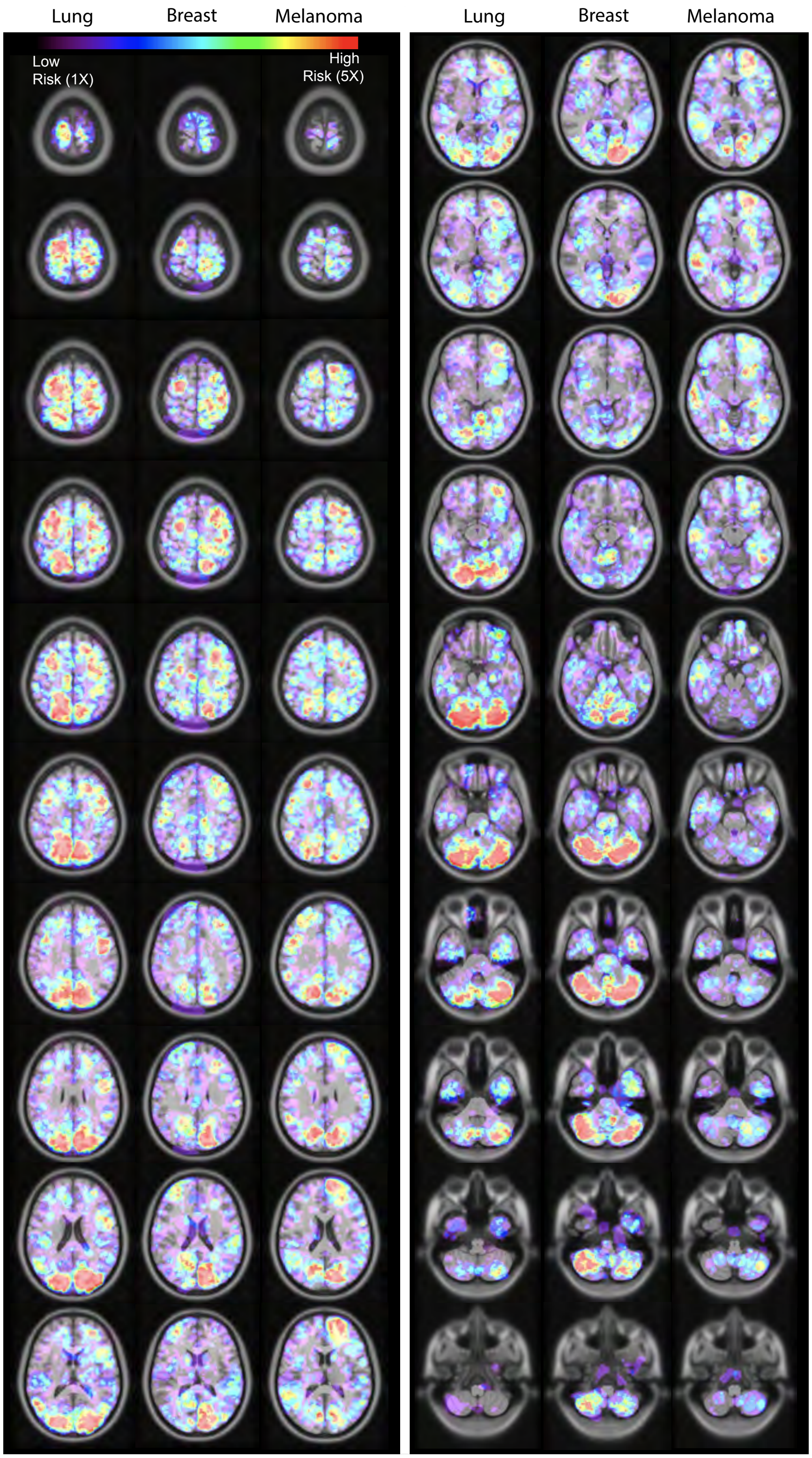
Lung
Breast
Melanoma
Lung
Breast
Melanoma
Low
Risk (1X)
High
Risk (5X)

Supplemental Figure 1. Brain metastasis spatial distribution comparison between lung, breast, and melanoma. Brain metastasis risk levels displayed on axial slices of the Montreal Neurological Institute (MNI) model for patients with lung (N=1,323 patients, L=5,324 lesions), breast (N=573 patients, L=3,078 lesions) and melanoma (N=409 patients, L=1996 lesions) primary cancer first diagnosis of brain lesions prior to stereotactic treatment.

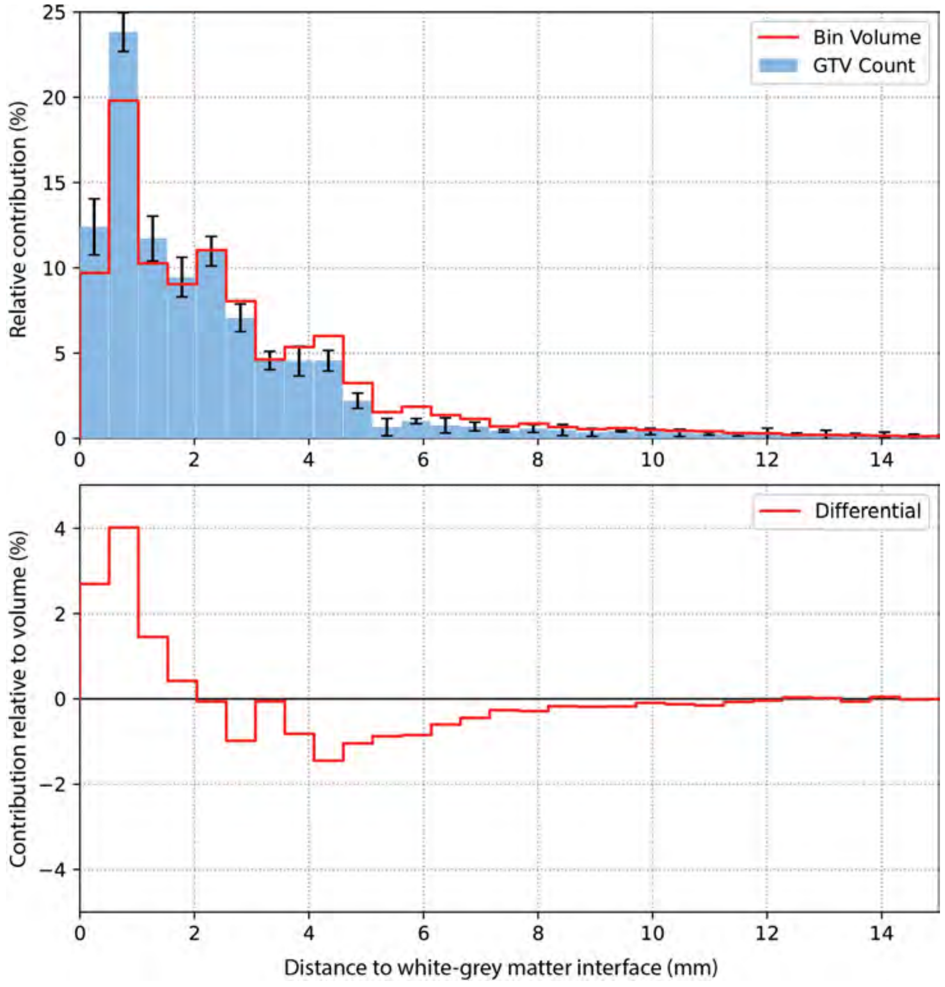

Supplemental Figure 2. Null-histogram analysis: brain metastasis occurrence versus the natural occurrence of convoluted brain tissue as a function of distance to the white and grey matter interface. Top – BM relative contribution as a function of distance to the white and grey matter interface. Bottom – Volume corrected contribution based on normal brain morphology as a function of distance to the white and grey matter interface. The observed spatial distribution of BMs is significantly closer ([with a 10% over-representation, P-value <0.05], Wilcoxon Signed Rank test) to the white-grey matter interface than expected under the null hypothesis of brain “fjords-liked” convoluted morphology. Source data are provided as a Source Data file.

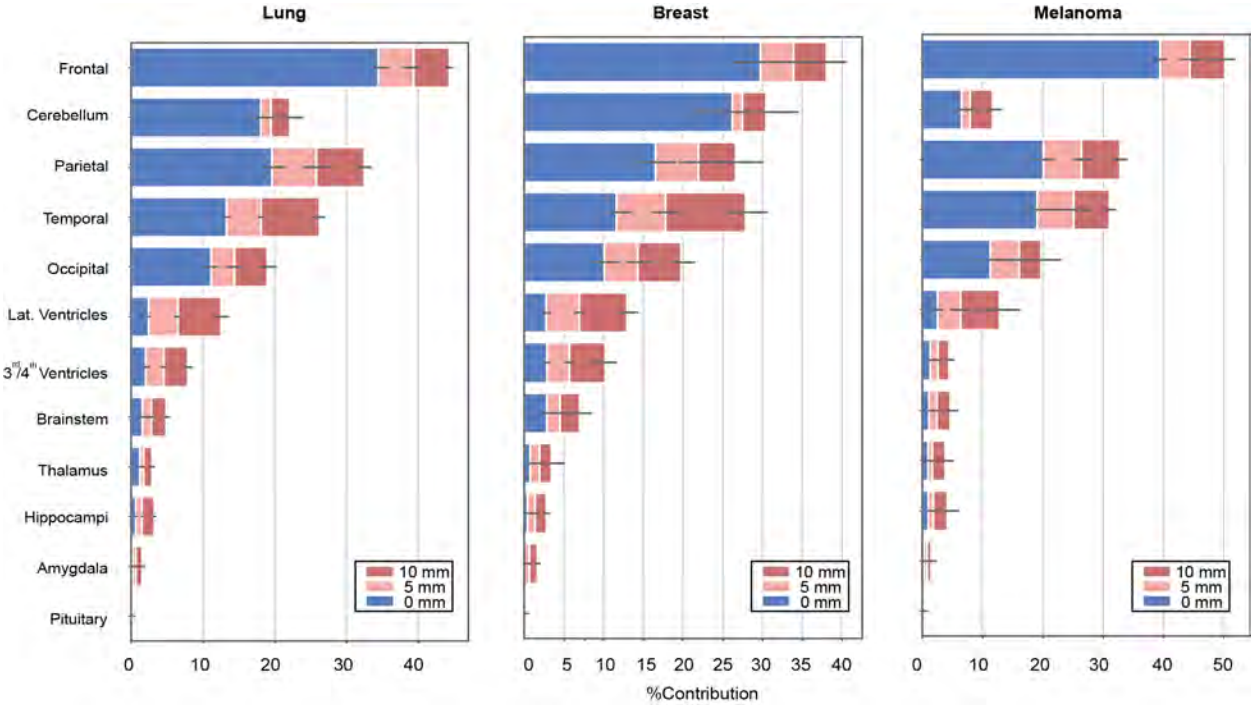

Supplemental Figure 3. Sensitivity analysis of the brain metastasis spatial distribution for different levels of regions expansions. Percentage BM contribution per anatomical coarse regions for 0-, 5- and 10-mm region expansions on Left – lung (N=1,315 patients, L=5,230 lesions), Middle – breast (N=570 patients, L=3,007 lesions), and Right – melanoma (N=407 patients, L=1931 lesions). Error bars indicate 95% confidence interval over the 4 institutions represented in the datasets. Source data are provided as a Source Data file.

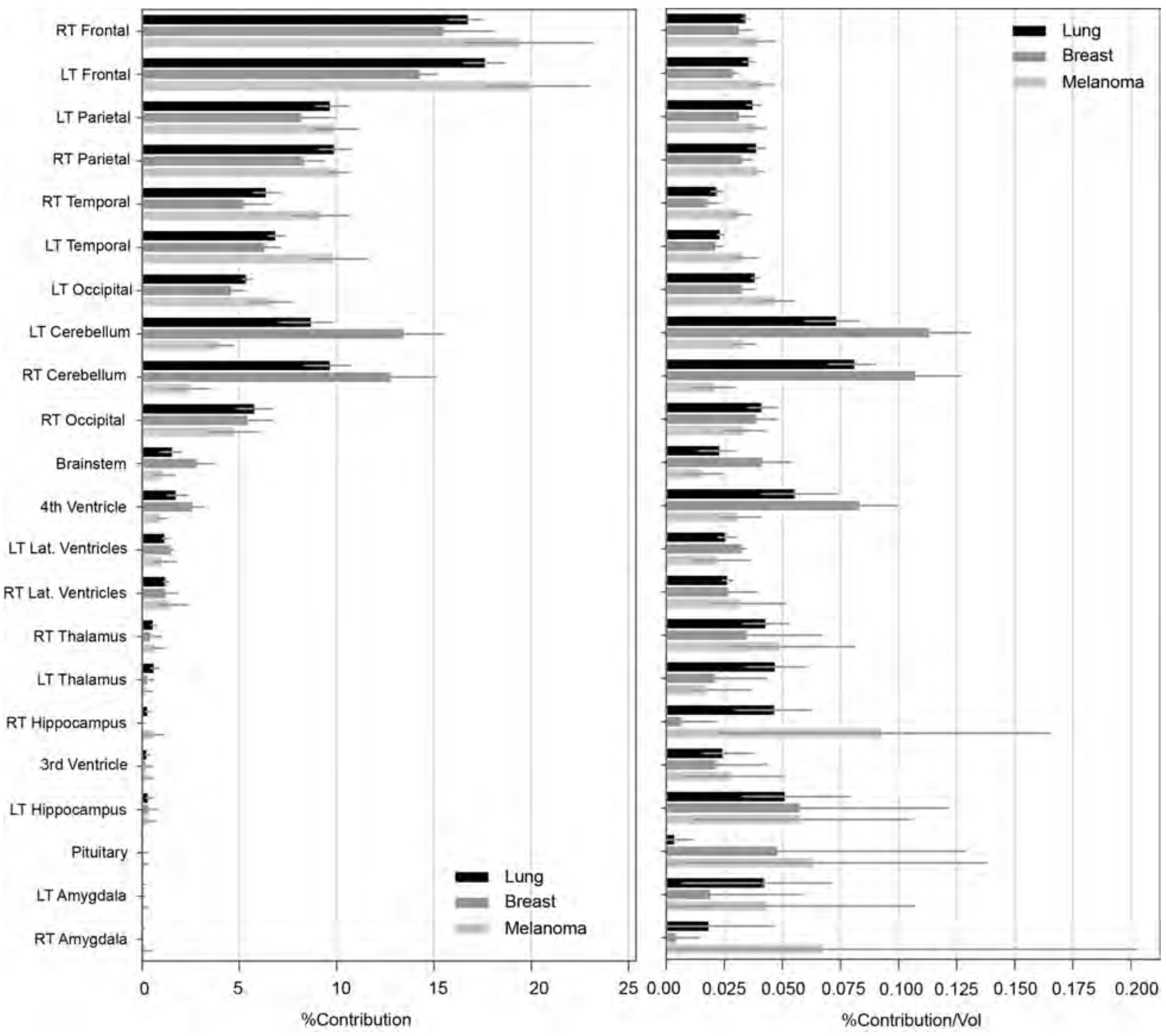

Supplemental Figure 4. Brain metastasis spatial distribution by laterality of coarse brain regions. Left - Percentage contribution of brain metastasis lesions by coarse right (RT) and left (LT) anatomical regions for lung (N=1,315 patients, L=5,330 lesions), breast (N=570 patients, L=3,007 lesions) and melanoma (N=407 patients, L=1931 lesions) primary cancer. Error bars indicate 95% confidence interval over the four institutions represented in the dataset. Right - Percentage contribution of brain metastasis normalized by the volume by right and left coarse anatomical regions for lung, breast and melanoma primary cancer. Error bars indicate 95% confidence interval over the four institutions represented in the dataset. Source data are provided as a Source Data file.

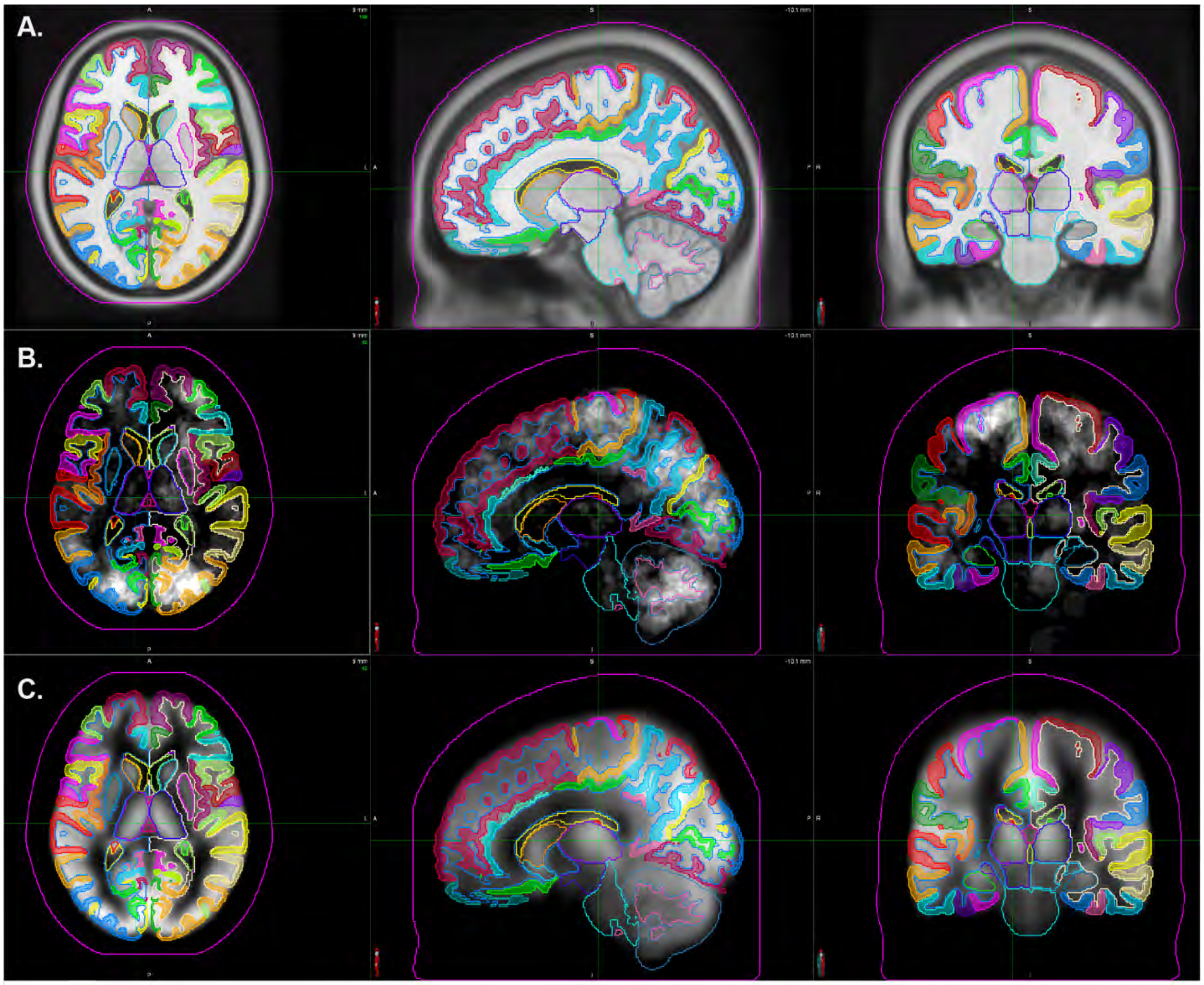

Supplemental Figure 5. Representative data of anatomical, computed brain metastasis risk map and perfusion in MNI space. Example of the Left - axial, Middle – sagittal, and Right - coronal anatomical fine [top] central slices aligned with the computed lung BM risk map (N=1,323 patients, L=5,324 lesions) [middle] and perfusion map in MNI space [bottom]. Tabular data available in Supplemental Table 1.

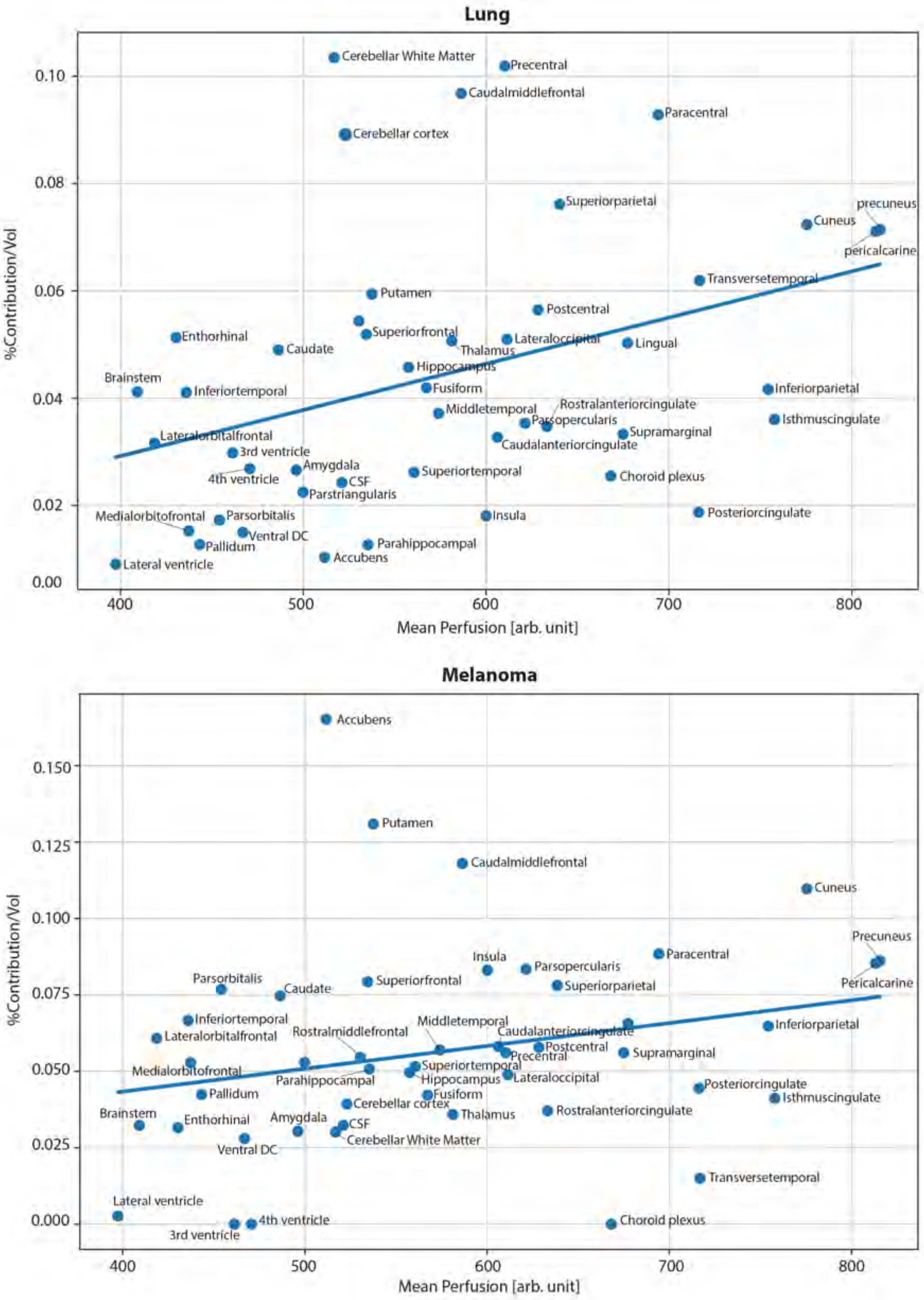
Lung
%Contribution/Vol
Mean Perfusion [arb. unit]
0.00
0.02
0.04
0.06
0.08
0.10
400
500
600
700
800
Cerebellar White Matter
Precentral
Caudalmiddlefrontal
Paracentral
Cerebellar cortex
Superiorparietal
precuneus
Cuneus
pericalcarine
Transversetemporal
Putamen
Postcentral
Superiorfrontal
Enthorhinal
Caudate
Thalamus
Lateraloccipital
Lingual
Hippocampus
Brainstem
Inferiortemporal
Fusiform
Inferiorparietal
Middletemporal
Rostralanteriorcingulate
Parsopercularis
Isthmuscingulate
Lateralorbitalfrontal
Supramarginal
Caudalanteriorcingulate
3rd ventricle
Amygdala
4th ventricle
Superiortemporal
Choroid plexus
CSF
Parstriangularis
Medialorbitofrontal
Parsorbitalis
Insula
Posteriorcingulate
Ventral DC
Pallidum
Parahippocampal
Accubens
Lateral ventricle
Melanoma
%Contribution/Vol
Mean Perfusion [arb. unit]
0.000
0.025
0.050
0.075
0.100
0.125
0.150
400
500
600
700
800
Accubens
Putamen
Caudalmiddlefrontal
Cuneus
Precuneus
Insula
Paracentral
Parsopercularis
Parsorbitalis
Superiorfrontal
Caudate
Superiorparietal
Pericalcarine
Inferiortemporal
Middletemporal
Inferiorparietal
Lateralorbitalfrontal
Rostralmiddlefrontal
Caudalanteriorcingulate
Postcentral
Precentral
Supramarginal
Medialorbitofrontal
Superiortemporal
Parahippocampal
Hippocampus
Lateraloccipital
Posteriorcingulate
Isthmuscingulate
Pallidum
Fusiform
Cerebellar cortex
Brainstem
Enthorhinal
Amygdala
CSF
Thalamus
Rostralanteriorcingulate
Ventral DC
Cerebellar White Matter
Transversetemporal
Lateral ventricle
4th ventricle
3rd ventricle
Choroid plexus

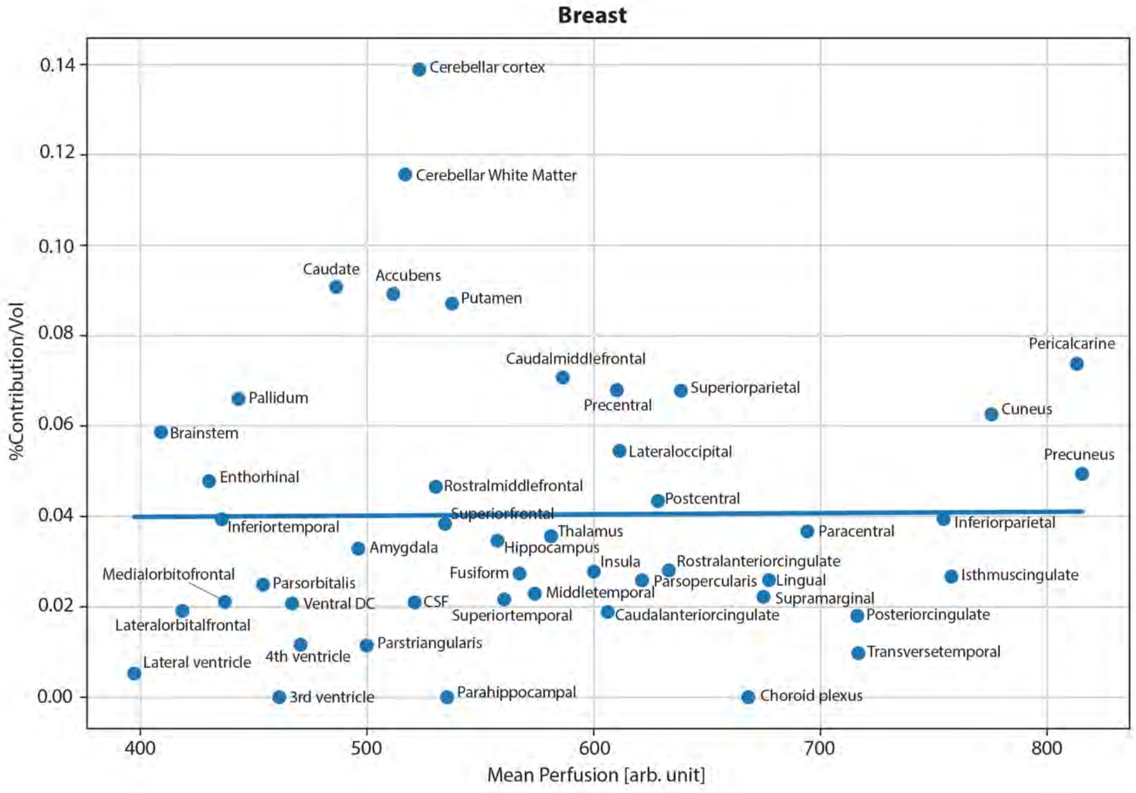

Supplemental Figure 6. Brain metastasis density versus mean perfusion per anatomical fine sub regions. Contribution per volume for all sub-regions of the fine anatomical atlas for Top – lung (N=1,323 patients, L=5,334 lesions), Middle – melanoma (N=409 patients, L=774 lesions), and Bottom – breast (N=573 patients, L=1,194 lesions) as a function of the mean perfusion of the regions in MNI space. Least-squares fit represented as the blue trend line. Source data are provided as a Source Data file.

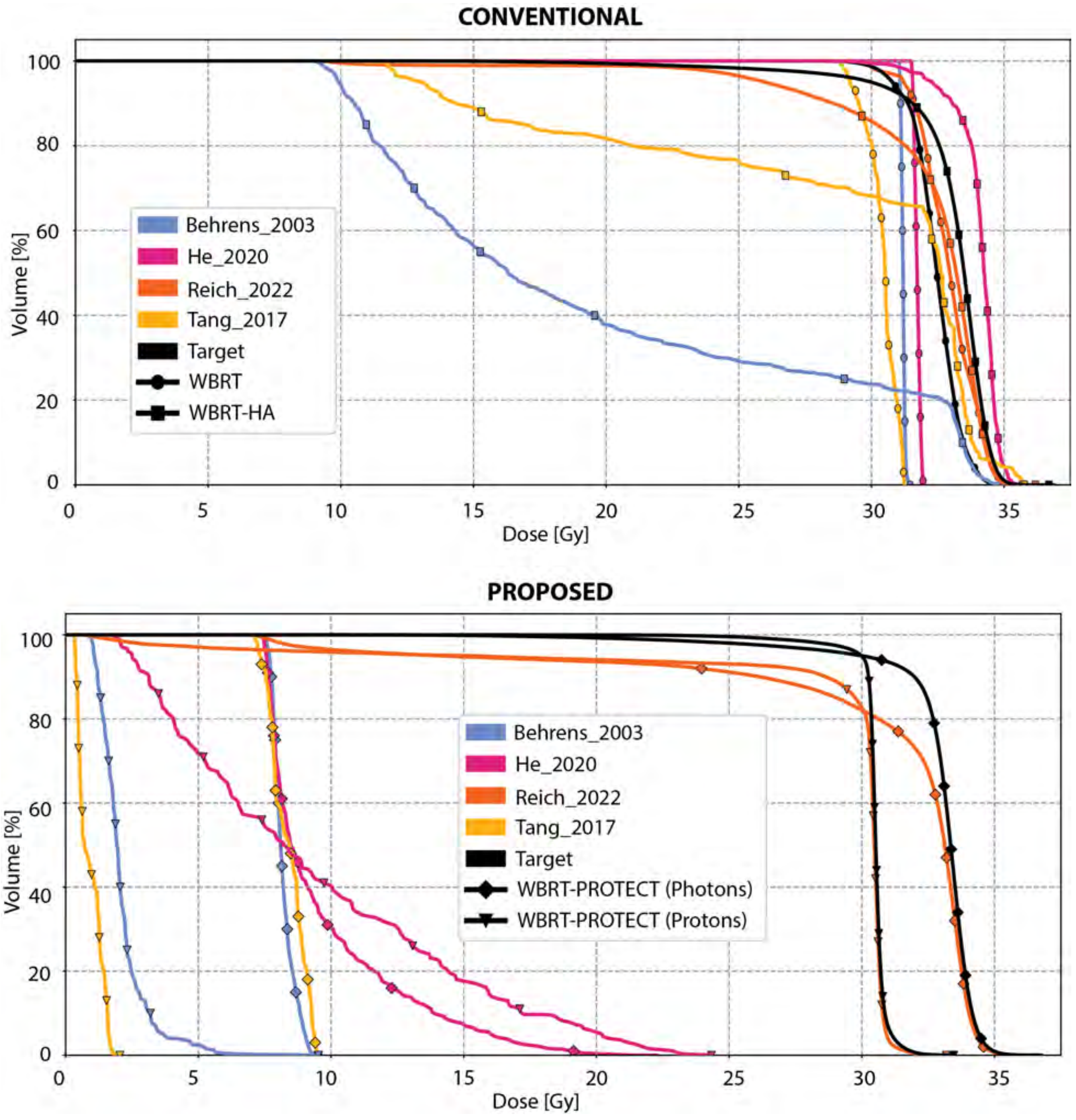

Supplemental Figure 7. Comparison of conventional and newly proposed WBRT techniques for functional sparing. Dose-volume histograms comparison of Top - The conventional techniques of WBRT and WBRT-HA, to Bottom – the newly proposed personalized approaches with higher functional sparing PROTECT (Personalized Radiation Optimization To Eliminate Collateral Toxicity) techniques planned using photons WBRT-PROTECT(Photons) or protons WBRT-

PROTECT(Protons). Dose volume histogram provided for target volume [black], and selected composite atlases Behrens 2003 [blue], He 2020 [magenta], Reich 2022 [orange], and Tang 2017 [yellow], using WBRT [circles], WBRT-HA [squares], WBRT-PROTECT(Photon) [diamonds], WBRT-PROTECT(Proton) [triangles]. Source data are provided as a Source Data file.

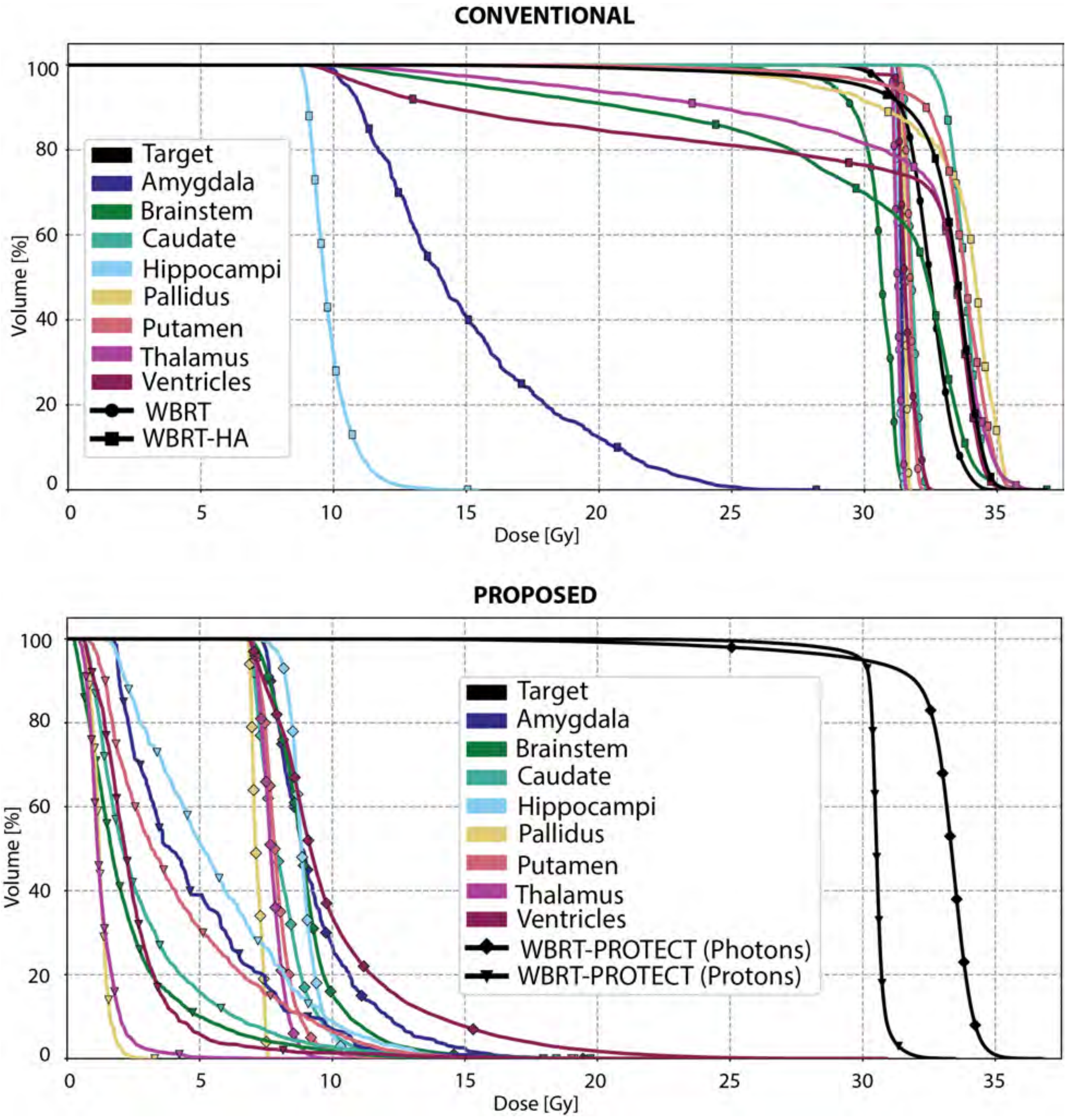

Supplemental Figure 8. Comparison of conventional and newly proposed WBRT techniques for anatomical sparing. Dose-volume histograms comparison of Top - The conventional techniques of WBRT and WBRT-HA to bottom – the newly proposed personalized approaches with higher anatomical sparing PROTECT (Personalized Radiation Optimization To Eliminate Collateral Toxicity) techniques planned using photons WBRT-PROTECT(Photons) or protons WBRT-

PROTECT(Protons). Target [black] and selected anatomical structures (hippocampi [light blue], amygdala [dark blue], brainstem [dark green], putamen [pink], caudate [light green], pallidus [yellow], thalamus [purple] and ventricles [dark purple]) using WBRT [circles], WBRT-HA [squares], and WBRT-PROTECT(Protons) [triangles]. Source data are provided as a Source Data file.

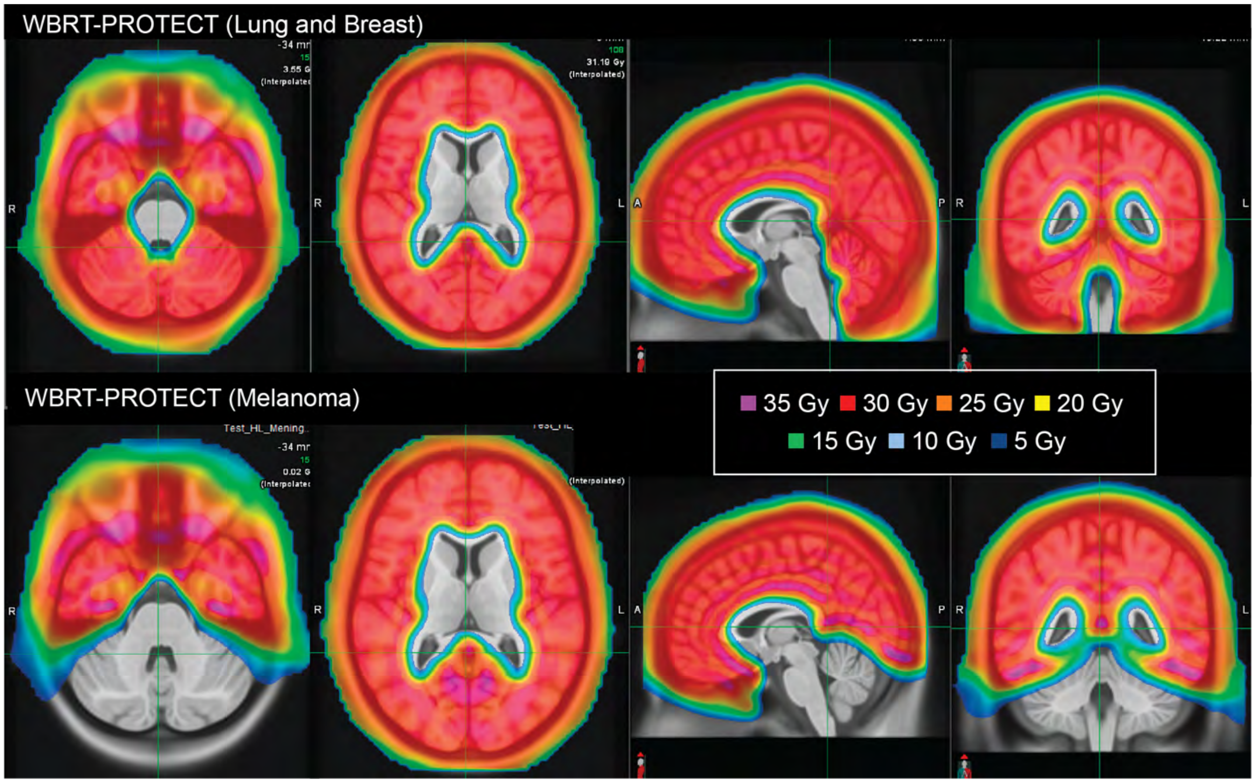

Supplemental Figure 9. Comparison of the proposed WBRT techniques for anatomical sparing based on the primary cancer histology. Possible examples of WBRT-PROTECT (Personalized Radiation Optimization To Eliminate Collateral Toxicity) proton treatment plans on representative axial, sagittal and coronal slices for top – lung and breast primary cancer, bottom -melanoma primary cancer.

Supplemental Table 1. Anatomical and functional region volume, mean ASL perfusion, percentage BM contribution and BM density for lung (N=1,323 patients, L=5,334 lesions), breast (N=573 patients, L=1,194 lesions) and melanoma (N=409 patients, L=774 lesions) primaries in MNI space.

REGION	VOLUME (cc)	MEAN PERFUSION	LUNG		BREAST		MELANOMA	
			Contribution (%)	Density (%/cc)	Contribution (%)	Density (%/cc)	Contribution (%)	Density (%/cc)
3rd Ventricle	1.26	461.32	0.04	0.03	0.00	0.00	0.00	0.00
4th Ventricle	2.80	470.68	0.08	0.03	0.03	0.01	0.00	0.00
Accumbens	1.82	511.61	0.02	0.01	0.16	0.09	0.30	0.17
Amygdala	4.94	496.16	0.13	0.03	0.16	0.03	0.15	0.03
Brain Stem	31.02	409.15	1.28	0.04	1.82	0.06	1.00	0.03
Caudal Anterior Cingulate	6.89	606.19	0.23	0.03	0.13	0.02	0.40	0.06
Caudal Middle Frontal	22.51	586.39	2.18	0.10	1.59	0.07	2.66	0.12
Caudate	10.74	486.40	0.53	0.05	0.97	0.09	0.80	0.07
Cerebellar Cortex	159.29	523.05	14.20	0.09	22.12	0.14	6.26	0.04
Cerebellar White Matter	41.57	516.89	4.30	0.10	4.81	0.12	1.25	0.03
Choroid Plexus	1.48	668.18	0.04	0.03	0.00	0.00	0.00	0.00
Cortical white matter	717.79	531.03	40.31	0.06	36.71	0.05	36.82	0.05
Cerebral Spinal Fluid	1.55	521.05	0.04	0.02	0.03	0.02	0.05	0.03
Cuneus	11.42	775.46	0.83	0.07	0.71	0.06	1.25	0.11
Entorhinal	4.76	430.34	0.24	0.05	0.23	0.05	0.15	0.03
Fusiform	23.73	567.31	1.00	0.04	0.65	0.03	1.00	0.04
Hippocampus	13.14	557.54	0.60	0.05	0.45	0.03	0.65	0.05
Inferior Lateral Ventricle	0.69	500.82	0.00	0.00	0.00	0.00	0.00	0.00
Inferior Parietal	37.89	754.33	1.58	0.04	1.49	0.04	2.45	0.06
Inferior Temporal	33.84	435.93	1.39	0.04	1.33	0.04	2.25	0.07
Insula	18.70	600.07	0.34	0.02	0.52	0.03	1.55	0.08
Isthmuscingulate	7.30	757.78	0.26	0.04	0.19	0.03	0.30	0.04
Lateral Ventricle	18.66	397.41	0.17	0.01	0.10	0.01	0.05	0.00
Lateral Occipital	32.79	611.41	1.67	0.05	1.79	0.05	1.60	0.05
Lateral Orbito-Frontal	25.55	418.61	0.81	0.03	0.49	0.02	1.55	0.06
Lingual	17.55	677.32	0.88	0.05	0.45	0.03	1.15	0.07
Medial Orbito-Frontal	12.33	437.31	0.19	0.02	0.26	0.02	0.65	0.05
Middle Temporal	43.97	574.04	1.63	0.04	1.01	0.02	2.51	0.06
Pallidum	5.91	443.34	0.08	0.01	0.39	0.07	0.25	0.04
Paracentral	14.17	694.25	1.31	0.09	0.52	0.04	1.25	0.09
Parahippocampal	5.93	535.36	0.08	0.01	0.00	0.00	0.30	0.05
Parsopercularis	13.82	621.31	0.49	0.04	0.36	0.03	1.15	0.08
Parsorbitalis	6.52	454.17	0.11	0.02	0.16	0.02	0.50	0.08
Parstriangularis	14.20	499.85	0.32	0.02	0.16	0.01	0.75	0.05
Pericalcarine	5.28	813.13	0.38	0.07	0.39	0.07	0.45	0.09
Post-Central	32.94	628.35	1.86	0.06	1.43	0.04	1.90	0.06
Posterior Cingulate	9.02	716.26	0.17	0.02	0.16	0.02	0.40	0.04
Pre-Central	40.18	610.20	4.09	0.10	2.73	0.07	2.25	0.06
Pre-Cuneus	30.25	815.44	2.16	0.07	1.49	0.05	2.61	0.09
Putamen	14.55	537.55	0.86	0.06	1.27	0.09	1.90	0.13
Rostral Anterior Cingulate	8.11	633.15	0.28	0.03	0.23	0.03	0.30	0.04
Rostral Middle Frontal	34.90	530.39	1.90	0.05	1.62	0.05	1.90	0.05
Superior Frontal	84.69	534.46	4.40	0.05	3.25	0.04	6.71	0.08
Superior Parietal	34.02	638.48	2.59	0.08	2.31	0.07	2.66	0.08
Superior Temporal	49.55	560.52	1.30	0.03	1.07	0.02	2.56	0.05
Supramarginal	32.15	674.88	1.07	0.03	0.71	0.02	1.80	0.06
Thalamus	23.72	581.20	1.20	0.05	0.84	0.04	0.85	0.04
Transverse Temporal	3.34	716.76	0.21	0.06	0.03	0.01	0.05	0.02
Ventral DC	12.53	466.92	0.19	0.01	0.26	0.02	0.35	0.03
WM Hypointensities	0.71	373.69	0.00	0.00	0.00	0.00	0.00	0.00
Other	---	---	0.02	---	2.37	---	2.25	---
