# Supplementary material for: Multi-institutional atlas of brain metastases informs spatial modeling for precision imaging and personalized therapy
Source: Nat Commun. 2025 May 15;16:4536. doi: 10.1038/s41467-025-59584-7 (PMC12081687; doi:10.1038/s41467-025-59584-7)
Supplement: Supplementary file 2 — Reporting Summary [file 41467_2025_59584_MOESM2_ESM.pdf]

nature portfolio

Corresponding author(s): Olivier Morin, PhD

Last updated by author(s): Mar 7, 2025

Reporting Summary

Nature Portfolio wishes to improve the reproducibility of the work that we publish. This form provides structure for consistency and transparency in reporting. For further information on Nature Portfolio policies, see our Editorial Policies and the Editorial Policy Checklist.

Statistics

For all statistical analyses, confirm that the following items are present in the figure legend, table legend, main text, or Methods section.

n/a	Confirmed	
☐	☒	The exact sample size (*n*) for each experimental group/condition, given as a discrete number and unit of measurement
☐	☒	A statement on whether measurements were taken from distinct samples or whether the same sample was measured repeatedly
☐	☒	The statistical test(s) used AND whether they are one- or two-sided<br>*Only common tests should be described solely by name; describe more complex techniques in the Methods section.*
☐	☒	A description of all covariates tested
☐	☒	A description of any assumptions or corrections, such as tests of normality and adjustment for multiple comparisons
☐	☒	A full description of the statistical parameters including central tendency (e.g. means) or other basic estimates (e.g. regression coefficient) AND variation (e.g. standard deviation) or associated estimates of uncertainty (e.g. confidence intervals)
☐	☒	For null hypothesis testing, the test statistic (e.g. *F*, *t*, *r*) with confidence intervals, effect sizes, degrees of freedom and *P* value noted<br>*Give P values as exact values whenever suitable.*
☒	☐	For Bayesian analysis, information on the choice of priors and Markov chain Monte Carlo settings
☐	☒	For hierarchical and complex designs, identification of the appropriate level for tests and full reporting of outcomes
☐	☒	Estimates of effect sizes (e.g. Cohen's *d*, Pearson's *r*), indicating how they were calculated

Our web collection on statistics for biologists contains articles on many of the points above.

Software and code

Policy information about availability of computer code

Data collection	The data collection process involved aggregating high-resolution, post-gadolinium contrast T1-weighted MRI scans and manually segmented brain metastases from 3,065 patients across four academic medical centers (UCSF, UCD, PMH, and MSKCC) between January 2011 and December 2023. A standardized clinical data entry template was used across institutions to ensure uniform patient demographic, tumor characteristic, and imaging data collection. Each MRI dataset was pre-processed, bias-corrected, co-registered to the Montreal Neurological Institute (MNI) anatomical space, and stored in a structured SQL database. The data processing pipeline, built using open-source tools, enabled rapid ingestion (170 studies per second) and standardization of imaging data, facilitating large-scale analysis of brain metastasis spatial distribution across anatomical, functional, and vascular brain regions.
Data analysis	The data analysis involved mapping 13,067 brain metastases onto the Montreal Neurological Institute (MNI) standard space to assess their spatial distribution across anatomical, functional, and vascular brain regions. Statistical analyses, including Welch's t-test and Wilcoxon signed-rank test, were performed to identify significant differences in metastasis density by cancer type, brain region, and perfusion level. Functional and vascular atlas overlays were used to examine metastasis occurrence in critical brain structures, revealing key sparing opportunities for personalized radiotherapy. A probabilistic model was developed to guide whole-brain radiotherapy (WBRT) optimization, balancing metastasis coverage while reducing radiation exposure to neurocognitive structures. Finally, treatment plans for function-sparing WBRT were simulated using photon and proton therapy techniques to assess potential reductions in radiation dose to protected brain regions. Code was written in python, SQL and jupyter notebooks. Code is available via the Open Science Framework (OSF) repository: https://osf.io/fkqmr/.

For manuscripts utilizing custom algorithms or software that are central to the research but not yet described in published literature, software must be made available to editors and reviewers. We strongly encourage code deposition in a community repository (e.g. GitHub). See the Nature Portfolio guidelines for submitting code & software for further information.

Data

Policy information about availability of data

All manuscripts must include a data availability statement. This statement should provide the following information, where applicable:

- Accession codes, unique identifiers, or web links for publicly available datasets
- A description of any restrictions on data availability
- For clinical datasets or third party data, please ensure that the statement adheres to our policy

The data sets that support the findings of this article are not publicly available due to reasonable privacy and security concerns. The underlying EHR data are not easily redistributable to researchers other than those engaged in the UCSF Institutional Review Board (IRB) approved for this study. However, access to de-identified data will be possible under a material transfer agreement (MTA) handled by the primary institution (UCSF). The data sets generated during and/or analysed during the current study are not publicly available for privacy reasons, but are available from the corresponding author on reasonable request.

Code is directly available from the following GitHub repository:
https://github.com/medomics/BrainMets_RiskMaps
Alternatively, code is available via the Open Science Framework (OSF) repository: https://osf.io/fkqmr/.

Research involving human participants, their data, or biological material

Policy information about studies with human participants or human data. See also policy information about sex, gender (identity/presentation), and sexual orientation and race, ethnicity and racism.

Reporting on sex and gender	Our reporting in Table 1 followed Nature Communications' policy on sex/gender.
Reporting on race, ethnicity, or other socially relevant groupings	Not applicable in this study.
Population characteristics	The detailed patients characteristics are summarized into Table 1.
Recruitment	All patients data used in this study were obtained from EHR, imaging and treatment planning database for patients treated for brain metastases using stereotactic radiosurgery.
Ethics oversight	The MEDomics effort obtained ethical approval for observational research using anonymized linked care data for supporting medical purposes that are in the interests of patients and the wider public. Specific efforts were approved by the Institutional Review Board (IRB) (UCSF: IRB# 20-32526, UCD: IRB# 2005950, PMH: REB CAPCR # 17-5662 and CAPCR# 18-5368 and MSKCC: IRB# 16-1307) and written informed consent for study inclusion was obtained from patients.

Note that full information on the approval of the study protocol must also be provided in the manuscript.

Field-specific reporting

Please select the one below that is the best fit for your research. If you are not sure, read the appropriate sections before making your selection.

[x] Life sciences [ ] Behavioural & social sciences [ ] Ecological, evolutionary & environmental sciences

For a reference copy of the document with all sections, see nature.com/documents/nr-reporting-summary-flat.pdf

Life sciences study design

All studies must disclose on these points even when the disclosure is negative.

Sample size	The largest number of patient data were included in each experiment based on data availability and 4 academic institutions.
Data exclusions	Exclusions were based on data completeness (and sufficien samples) according to the relevant criteria established in the paper for each experiment.
Replication	Multiples independent experiments/replicates were performed. All attempts at replication were successful and results were consistent with our primary findings. All experiments were reproducible. Details on replicates and sample size are described in the paper for each case.
Randomization	Since our study is a retrospective analysis of routine care data from EHR, no randomization in samples allocation was used. Error bars are present in all analyses of relevance.
Blinding	Since our study is a retrospective analysis of routine care data from EHR and treatment planning systems, blinding was not applicable.

Reporting for specific materials, systems and methods

We require information from authors about some types of materials, experimental systems and methods used in many studies. Here, indicate whether each material, system or method listed is relevant to your study. If you are not sure if a list item applies to your research, read the appropriate section before selecting a response.

Materials & experimental systems

n/a	Involved in the study
☒	☐ Antibodies
☒	☐ Eukaryotic cell lines
☒	☐ Palaeontology and archaeology
☒	☐ Animals and other organisms
☐	☒ Clinical data
☒	☐ Dual use research of concern
☒	☐ Plants

Methods

n/a	Involved in the study
☒	☐ ChIP-seq
☒	☐ Flow cytometry
☒	☐ MRI-based neuroimaging

Clinical data

Policy information about clinical studies

All manuscripts should comply with the ICMJEguidelines for publication of clinical research and a completedCONSORT checklist must be included with all submissions.

Clinical trial registration	In this study, we used real-world retrospective patients data and no clinical trials was conducted. Data was collected at four academic institutions.
Study protocol	No clinical trials was conducted for this study. This retrospective study utilized routine clinical care data from EHR, imaging and DICOM-RT from treatment planning systems.
Data collection	We did not collect our own data for this study. This retrospective study utilized routine clinical care data from EEHR, imaging and DICOM-RT from treatment planning systems.
Outcomes	We did not use clinical outcomes in this study.

Plants

Seed stocks	*Report on the source of all seed stocks or other plant material used. If applicable, state the seed stock centre and catalogue number. If plant specimens were collected from the field, describe the collection location, date and sampling procedures.*
Novel plant genotypes	*Describe the methods by which all novel plant genotypes were produced. This includes those generated by transgenic approaches, gene editing, chemical/radiation-based mutagenesis and hybridization. For transgenic lines, describe the transformation method, the number of independent lines analyzed and the generation upon which experiments were performed. For gene-edited lines, describe the editor used, the endogenous sequence targeted for editing, the targeting guide RNA sequence (if applicable) and how the editor was applied.*
Authentication	*Describe any authentication procedures for each seed stock used or novel genotype generated. Describe any experiments used to assess the effect of a mutation and, where applicable, how potential secondary effects (e.g. second site T-DNA insertions, mosiacism, off-target gene editing) were examined.*
